# Liquid Phase Electron Microscopy of Bacterial Ultrastructure

**DOI:** 10.1002/smll.202402871

**Published:** 2024-09-06

**Authors:** Brian J. Caffrey, Adrián Pedrazo‐Tardajos, Emanuela Liberti, Benjamin Gaunt, Judy S. Kim, Angus I. Kirkland

**Affiliations:** ^1^ The Rosalind Franklin Institute Harwell Science and Innovation Campus Didcot OX11 OQX UK; ^2^ Nuffield Department of Women's & Reproductive Health University of Oxford John Radcliffe Hospital Oxford OX3 9DU UK; ^3^ Department of Materials University of Oxford Oxford OX1 3PH UK

**Keywords:** deinococcus.radiodurans, energy dispersive x‐ray spectroscopy, graphene encapsulation, liquid phase, manganese uptake, radiation resistance, scanning transmission electron microscopy

## Abstract

Recent advances in liquid phase scanning transmission electron microscopy (LP‐STEM) have enabled the study of dynamic biological processes at nanometer resolutions, paving the way for live‐cell imaging using electron microscopy. However, this technique is often hampered by the inherent thickness of whole cell samples and damage from electron beam irradiation. These restrictions degrade image quality and resolution, impeding biological interpretation. Using graphene encapsulation, scanning transmission electron microscopy (STEM), and energy‐dispersive X‐ray (EDX) spectroscopy to mitigate these issues provides unprecedented levels of intracellular detail in aqueous specimens. This study demonstrates the potential of LP‐STEM to examine and identify internal cellular structures in thick biological samples. Specifically, it highlights the use of LP‐STEM to investigate the radiation resistant, gram‐positive bacterium, *Deinococcus radiodurans* using various imaging techniques.

## Graphical abstract

Imaging whole live biological specimens in their native aqueous environments at sub‐cellular resolutions is of great importance to the understanding of fundamental biological interactions. Using scanning transmission electron microscopy (STEM), energy dispersive X‐ray spectroscopy (EDX) and graphene encapsulation methods, the elemental distribution and ultrastructural detail of *D.radiodurans* is revealed in liquid, an alternative to the thin lamellar sections of cryo‐tomography.

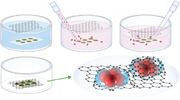

## 1 Introduction

Liquid phase electron microscopy (LP‐EM) is a technique that enables the analysis of intracellular, real‐time dynamics with nanometer precision in native, aqueous environments. This emerging field of research has provided unique insights into the dynamic processes of hard materials,^[^ ^1^ ^]^ such as the formation of platinum nanocrystals^[^ ^2^ ^]^ and electrochemical studies of lithium‐ion batteries.^[^ ^3^ ^]^ More recently, LP‐EM has also expanded to the analysis of biological materials such as proteins,^[^ ^4^ ^]^ bacteria,^[^ ^5^ ^]^ yeast^[^ ^6^ ^]^ and human cells.^[^ ^7^, ^8^ ^]^ However, ultrastructural study of the internal components of these biological materials is difficult due to limitations imposed by sample thickness and electron beam fluence.

Central to addressing these limitations are considerations of the most appropriate geometry for LP‐EM imaging of biological samples. Conventional transmission electron microscopy (TEM) is widely used in biological LP‐EM^[^ ^9^ ^]^ and in structural biology more generally.^[^ ^10^ ^]^ The popularity of brightfield TEM (BF‐TEM) in biological sciences is due, in part, to phase contrast being dominant for low atomic number soft matter samples.^[^ ^11^ ^]^ However, spatial resolution in BF‐TEM decreases significantly beyond thicknesses greater than the elastic mean free path of electrons in water (*λ*≈300 nm at 300 kV).^[^ ^12^ ^]^ In addition, as the sample thickness increases the fluence in the elastically scattered wavefield decreases, reducing the phase contrast even for zero‐loss filtered images. As most prokaryotic and eukaryotic cells are greater than 500 nm in thickness, conventional BF‐TEM therefore has inherent limitations for high‐resolution imaging of whole cell samples at intermediate voltages. Annular darkfield STEM (ADF‐STEM) which records incoherently scattered electrons, can provide superior spatial resolution as it is less affected by chromatic aberration,^[^ ^13^ ^]^ although at the expense of a reduced signal due to weak scattering at high angles from light element materials. The detection geometry used for ADF‐STEM can also provide valuable complementary information on the elemental composition of biological samples using energy dispersive X‐ray (EDX) spectroscopy which, for example, has been used for the localization of biominerals with distinct elemental signatures in bacteria^[^ ^14^ ^]^ and in tissue sections to distinguish between different heavy metal conjugated immuno‐labels.^[^ ^15^ ^]^

While ADF‐STEM gives improved image contrast compared to BF‐TEM for thicker samples, the total thickness of the liquid cell in which the specimen is enclosed plays a significant role in limiting the achievable resolution and signal‐to‐noise ratios (SNR) of both LP‐ADF‐STEM imaging and EDX spectroscopy.^[^ ^16^ ^]^ This sample thickness limitation is compounded further by the need for samples to be enclosed in thin membrane windows to partition the sample from the microscope vacuum. For instance, each silicon nitride (Si_3_N_4_) window, above and below the specimen in dedicated TEM liquid holders is typically ≈50 nm thick, in addition to the liquid/sample thickness. Graphene liquid cells (GLC) overcome some of these limitations by reducing the liquid encapsulation to a single atomic layer either side of the sample, therefore maximizing the imaging and spectral resolution from aqueous environments in LP‐EM applications.

The selection of appropriate biological models is also important to achieving high‐resolution images using LP‐EM. The *Deinococcus* genus is distinguished by an extraordinarily high tolerance to the effects of environmental hazards, such as arsenic,^[^ ^17^ ^]^ ionizing and UV irradiation,^[^ ^18^ ^]^ and desiccation.^[^ ^19^ ^]^ *D. radiodurans*, the type species of the genus, is a spherical, non‐motile, didermal bacterium and is one of the most radiation‐resistant of the *Deinococcus* genus and thirty times more resistant than the more commonly studied, *Escherichia coli*.^[^ ^20^ ^]^ While the biochemical mechanisms underlying this resistance are not yet fully understood, it is thought that this extreme radiation resistance has evolved from its adaptation to dry environments^[^ ^21^ ^]^ with dehydration being a more common physiological stressor than environmental radiation. Mechanisms for the unusual radiation resistance of *D.radiodurans* have been suggested to involve DNA damage resistance and repair pathways, as evidenced by the unusually large number (>100) of radiation‐induced double‐strand breaks (DSBs) that this species can withstand without lethality or mutagenesis. The proteins responsible for these resistance and repair pathways require protection from the oxidative environments created by irradiation and are therefore highly dependent on radical oxygen scavenger Mn^2+^ complexes^[^ ^22^ ^]^ and antioxidant carotenoids such as deinoxanthin.^[^ ^23^ ^]^ In turn, these chemicals require a rich source of nutrients to maintain effective concentrations. These resistance mechanisms may allow *D.radiodurans* to tolerate electron irradiation for longer, mitigating the effects of electron beam damage.^[^ ^24^ ^]^ *D.radiodurans* is therefore an ideal candidate for demonstrating ultrastructural study using LP‐EM techniques.

In this work, we demonstrate the capabilities of graphene encapsulation and STEM‐EDX imaging in studies of biological specimens in the liquid phase and use these to provide insights into *D.radiodurans* ultrastructure. We show the localization of manganese ions in phosphate‐containing storage granules within the bacterium and demonstrate the ultrastructural response of *D.radiodurans* to nutrient restriction and desiccation in situ.

## 2 Results

### 2.1 Graphene Liquid Cell Assembly and Morphology of *D.radiodurans*

**Figure 1:**
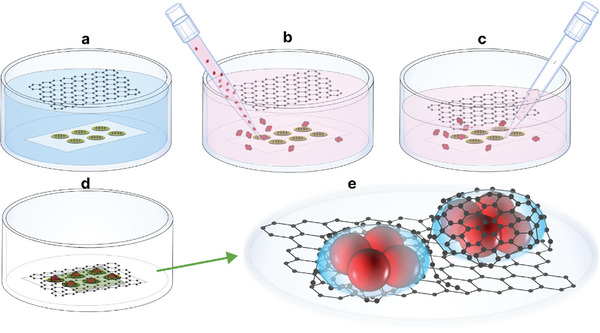
Schematic of graphene liquid cell (GLC) assembly and LM/EM analysis of encapsulated *D.radiodurans*. a) Freshly prepared single‐layer graphene grids placed in water on filter paper, with the second layer of graphene suspended above. b) After exchanging water with buffer (HEPES), the sample is added directly to the top of the graphene grids. c) The solution is removed slowly to ensure the graphene layer remains intact over the grids. d) Solution is completely removed, and grids allowed to air dry for GLC comprised of multiple blisters. e) Expanded view of the panel (d) of graphene blisters with encapsulated *D.radiodurans*.

Using the graphene encapsulation method developed previously,^[^ ^25^, ^26^ ^]^ it is possible to confine biological samples within graphene encapsulated aqueous environments using standard Au‐flat and Au Quantifoil grids (**Figures** **1** and S1, Supporting Information). In this work, a single‐layer graphene grid was submerged in a container with a biological buffer of interest and the sample was pipetted over it. The second layer of graphene was then carefully lowered onto the grid by removing excess buffer from the container and the sealed GLC is allowed to dry. The encapsulated bacteria were immediately inspected using brightfield light microscopy (LM). The graphene encapsulation appears to be tolerated by *D.radiodurans* as shown by the integrity of the membrane under visual inspection in LM using trypan blue and directly in EM with no vacuum‐associated shrinking^[^ ^27^ ^]^ (Figures S2a–d and S3a–d, Supporting Information). By measuring cell size from acquired ADF‐STEM images (Figure S4, Supporting Information), we calculated the approximate value of the sustained pressure on *D.radiodurans* cells using the Laplace pressure,^[^ ^28^ ^]^ yielding an approximate pressure of 0.5 atmospheres (≈50 kPa). A more detailed methodology including different pressure contributions such as the Van der Waals and elastic components,^[^ ^29^ ^]^ yields a pressure of 1.6 atmospheres (≈162 kPa) (Figure S5, Supporting Information). This result is in good agreement with both the previous membrane observations^[^ ^27^ ^]^ and the calculated pressure for samples as thick as bacteria or cells (1 µm) using different approaches.^[^ ^28^ ^]^ In the case that direct contact between the pristine graphene and the hydrated membrane of *D.radiodurans* is considered, the estimated pressure is several orders of magnitude lower (See analysis in Supporting Information for more details). In all lysogeny broth (LB) cultures, *D.radiodurans* cells typically formed tightly packed tetrads, with a high degree of asymmetric division and poorly formed central septa in early exponential growth phases and predominantly octad or higher order assemblies with thick central septa in stationary phase cultures (Figure S3a–d, Supporting Information). In low nutrient media preparations, the cells primarily formed single cocci or diads, as observed previously in studies of *D.radiodurans* nutrient‐dependent pleomorphisms.^[^ ^30^, ^31^ ^]^

### 2.2 *D.radiodurans* Growth Stages, Ultrastructure, and Elemental Distribution

Imaging whole bacteria provides cellular context to subcellular features, allowing the screening of multiple bacteria at different stages of growth and capturing rare cellular events which may be obscured by sample thickness or removed by thinning for conventional cryo‐EM studies.^[^ ^32^ ^]^

**Figure 2:**
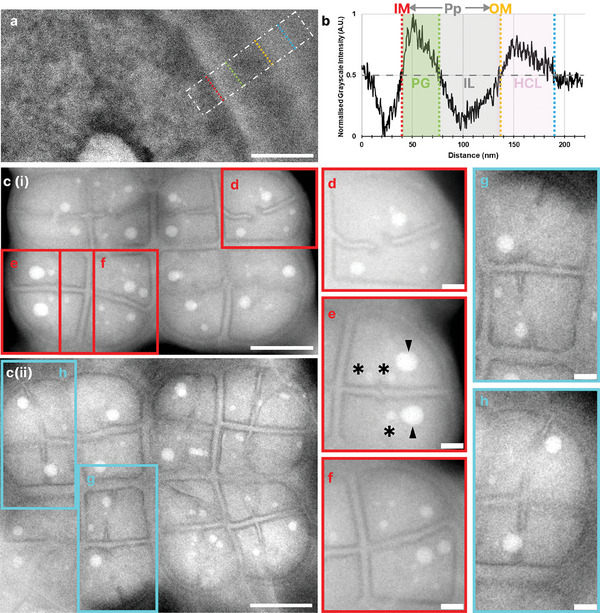
*D.radiodurans* growth stages and membrane composition in graphene encapsulated environments. a) Bandpass (BP) filtered LP‐ADF‐STEM section of encapsulated late exponential phase (OD_600_: 0.65) *D.radiodurans* cell envelope, with a density profile region of interest (ROI) highlighted as white dashed box (TCF: 37500 e^−^/nm^2^). b) Corresponding density profile of cell envelope components reveals a periplasmic space (Pp) between the inner and outer membrane, a peptidoglycan layer (PG) followed by an interstitial layer (IL) and a final high contrast layer (HCL). c i, ii) BP filtered LP‐ADF‐STEM images of encapsulated stationary phase *D.radiodurans* octads at various stages of division from the same sample (TCF: 93 e^−^/nm^2^). d–h): Expanded view of single cocci from octad ROIs showing septum progression stages, rotated to place the central septum in the bottom left of the ROIs. d) Characteristic septal aversions visible. e) Septa meeting side on, in the cell center. Asterisk (*): Small storage granules; Arrowheads: Phosphate storage granules. f) Two cocci separated by a completed septum. g) New septa begin to emerge perpendicular to the central septum. h) Later stage of tetrad formation. Scale bar: a: 100 nm; c i, ii: 1 µm, d–h: 250 nm.

Bacteria were encapsulated at different phases of the bacterial growth curve; with multiple high contrast granules present in the stationary phase. The periplasmic space between the outer and inner membrane (OM and IM) was ≈90 nm (**Figure** **2**a,b), with a peptidoglycan (PG) component ≈35 nm thick and an interstitial layer (IL) ≈55 nm thick. This agrees with previously reported descriptions of a dense diffuse periplasmic layer^[^ ^33^ ^]^ and cell envelope thickness measurements from frozen vitrified sections^[^ ^34^ ^]^ of *D.radiodurans*. The surface layer (S‐layer), typically reported as a 20 nm thick layer,^[^ ^34^ ^]^ was not clearly visible. However, a high contrast layer (HCL), ≈55 nm thick, likely containing both the S‐layer and a carbohydrate coat^[^ ^35^, ^36^, ^37^, ^38^ ^]^ was observed (Figure **2**a,b).

The characteristic stages of septal growth and cell division in *D.radiodurans* were observed in encapsulated bacteria (Figure 2ci,ii). Septal growth begins perpendicular to the central septum of the tetrad, dividing a single coccus into two cocci with characteristic septal aversion, as previously reported in conventional EM studies (Figure 2d,e).^[^ ^39^ ^]^ Large phosphate granules were evident in the majority of *D.radiodurans* cocci, with smaller storage granules ranging from 40 to 150 nm in size, potentially used for carbohydrate storage often also visible,^[^ ^40^ ^]^ as highlighted in Figure 2e. In early exponential cultures, the phosphate granules appeared smaller, were difficult to differentiate from carbohydrate granules and were also more numerous (Figure S3a, Supporting Information). Merging of the septa leads to complete cellular division into two cocci (Figure 2f). Further division into four cocci begins with the formation of septa in the center of and perpendicular to the previous septum and from opposite sides of the cell envelope (Figure 2g). The septa advance from the central and peripheral edges toward the center of the coccus with a diminishing slit formed between the advancing septal edges (Figure 2h), as observed previously by optical microscopy^[^ ^41^ ^]^ and conventional EM.^[^ ^39^ ^]^

Several asymmetric divisions of cocci were also observed (Figure S6a, Supporting Information), along with s‐shaped septal growths from the center of the cell (Figure S6b,c, Supporting Information) and multiple diagonally orientated septa at various stages of division (Figure S6d,e, Supporting Information). It was possible to image these structures at fluences as low as ≈0.5 e^−^/nm^2^ (Figure S7, Supporting Information). A fluence of 0.5 e^−^/nm^2^ is an order of magnitude lower than the fluence threshold for maintaining transcriptionally active *E.coli*, suggesting live cell imaging of these structures may be possible in the near future.^[^ ^42^ ^]^ Note, while the terms electron dose and electron fluence are often used interchangeably in the field of electron microscopy, electron fluence is defined as the number of impacted electrons on a sample per unit area, i.e., 100 e^−^/nm^2^ = 1 e^−^/Å^2^, and electron dose is strictly defined as the energy absorbed per unit area, expressed in units of Grays. Therefore, in all subsequent images, we quote the total cumulative fluence (TCF) in SI units of e^−^/nm^2^, which accounts for all electron beam irradiation up to and including the image shown.

**Figure 3:**
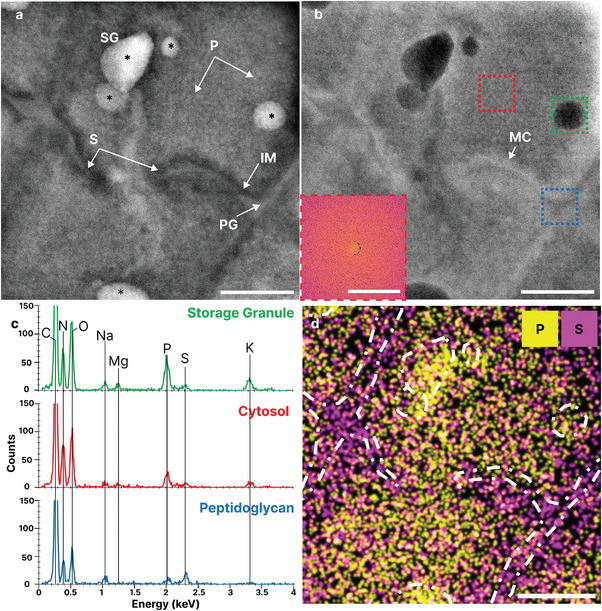
Ultrastructural detail and elemental distribution in *D.radiodurans* using LP‐ STEM and EDX. a) BP filtered LP‐ADF‐STEM and b) LP‐BF‐STEM image of encapsulated bacteria internal structure (TCF: 11800 e^−^/nm^2^), **Inset**: Expanded PS of cytosolic (red) ROI, with spatial frequency at (18 nm^−1^) marked (dashed red). c) EDX spectra from ROIs in (b). d) Gaussian filtered and contrast normalized phosphorous and sulfur EDX maps of (a,b) (TCF: 50300 e^−^/nm^2^). Dashed white lines: Outline of membrane and polyphosphates from (a). Scale bar for a, b, d: 250 nm; PS: (2 nm^−1^).

At higher magnifications, additional ultrastructural features were visible within the encapsulated cells (**Figures** **3**a and S8a–d, Supporting Information), including protein (P), peptidoglycan (PG), septal edges (S), and storage granules (SG) (Black asterisk; Figure 3a). Protein was dispersed throughout the cell and power spectra (PS) calculated from image areas containing protein (Figure 3b, red ROI), show enhanced frequencies corresponding to a spacing of 24 ± 8 nm consistent with bacterial ribosomes, not observed in the PS calculated from the peptidoglycan region, blue ROI (Figure 3b, Inset; Figure S8e–h, Supporting Information).

EDX analysis of the IM and PG indicates a relatively high abundance of sulfur. We propose that this periplasmic layer may act as a reservoir for sulfur‐containing amino acids and low molecular weight thiols, such as cysteine/methionine and bacillithiol, respectively, which are integral to the cells redox homoeostasis and antioxidant defense system.^[^ ^43^, ^44^, ^45^ ^]^ Most storage granules imaged were enriched in phosphorus, oxygen, and potassium (Figures 3c and S9, Supporting Information). Other counterions, including magnesium, were also observed to correlate spatially with phosphate granules although the EDX SNR was poor due to the relatively low fluence used for acquisition. While calcium counterions were also observed in polyphosphates with *D.radiodurans*, these were rare and usually found only in single polyphosphate granules within desiccated individuals. Sulfur was found to correlate with the cell membrane, along with sodium (S9, Supporting Information).

**Figure 4:**
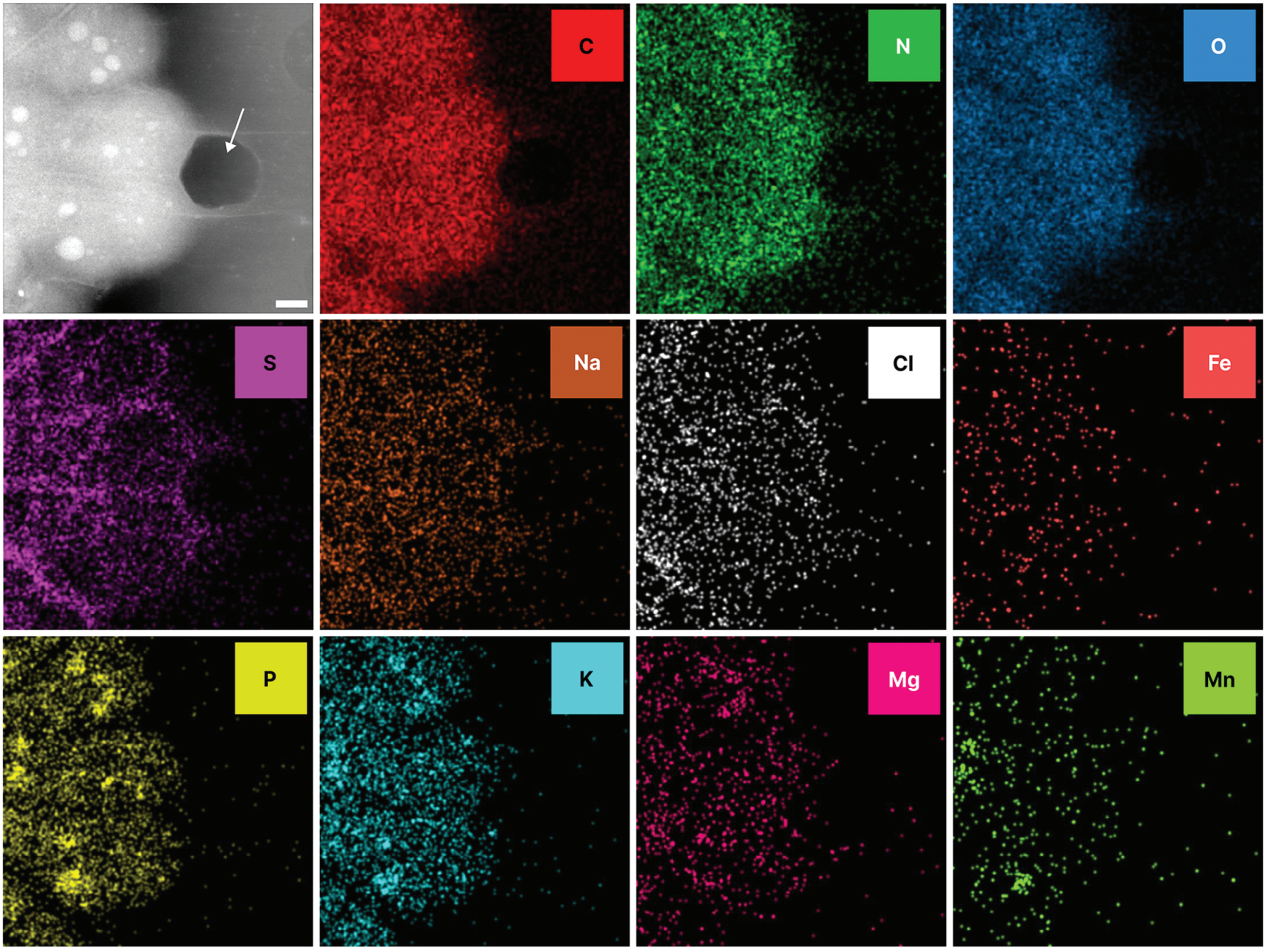
Manganese accumulates in bacterial phosphate granules. Summed LP‐ADF‐STEM image of bacteria incubated in MnCl_2_ (Average TCF: 2979 e^−^/nm^2^); White Arrow: Gaseous bubble. **Top Row**: Intracellular elements; **Middle row**: Cell membrane associated elements; **Bottom Row**: Polyphosphate associated elements. Scale bar: 500 nm.

Spherical, low contrast particles were also observed, ≈30 nm in size, associated with the IM on the cytoplasmic side and are proposed to be similar structures to those previously reported as macromolecular complexes (MC),^[^ ^34^ ^]^ thought to be Dps2 clusters for iron sequestration^[^ ^22^ ^]^ (White arrow; Figure 3b). However, there was no significant iron signal found anywhere within the cell in the EDX maps (Figure S9, Supporting Information). This is possibly due to iron being dispersed throughout the cell rather than being concentrated in any specific area making the signal extremely weak in an already relatively low signal map (Figure S9b, Supporting Information).

### 2.3 Manganese Accumulates in Bacterial Phosphate Granules

Manganese is implicated in *D.radiodurans* radiation resistance and appears to localize with phosphate ions^[^ ^22^ ^]^ suggesting that manganese acts as a counterion to cellular phosphate. Using EDX analysis, at biologically relevant fluences, i.e., <3000 e^−^/nm,^2[^ ^46^ ^]^ we confirm that manganese localizes to phosphate granules following MnCl_2_ supplementation of bacterial media (**Figures** 4 and S10a, Supporting Information). However, we were unable to observe accumulations of naturally occurring manganese‐containing polyphosphates in *D.radiodurans* at the fluences used to record the EDX maps (Figure S10b,c, Supporting Information). Interestingly, manganese is not distributed uniformly across all polyphosphates within the cell, suggesting distinct biological roles for the different polyphosphate‐cation identities. These manganese‐containing phosphate granules may act as a store of reactive oxygen species‐scavenging manganese cations for release during oxidative stress.

### 2.4 Vacuum Desiccation in *D.radiodurans* Leads to Separation of Cell Membrane, a Marker for Liquid Encapsulation

**Figure 5:**
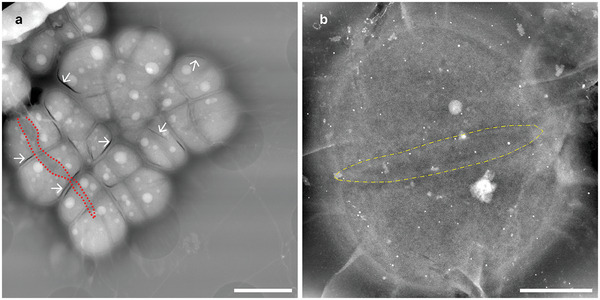
Effect of different environmental stressors on *D.radiodurans* ultrastructure. a) LP‐ADF‐STEM image of vacuum desiccated bacteria in a torn graphene liquid cell (TCF: 93 e^‐^/nm^2^); Dashed red line: Tear in graphene, White arrows: Examples of desiccation‐related membrane separation. b) LP‐ADF‐STEM image of encapsulated diad, grown in 10% LB media (TCF: 2334 e^‐^/nm^2^); Dashed yellow line: Ring shaped membrane between individuals. Scale bar for a: 2 µm, b: 500 nm.

To assess the effect of vacuum desiccation on *D.radiodurans*, a sample of exponentially growing bacteria was placed in a graphene grid as previously mentioned, however after a brief period (<5 mins) of air drying the outer grid, it was placed directly into the microscope. Bacteria exposed to the microscope vacuum detached from their membranes causing large cracks to form (**Figures** **5**a and S11a–d, Supporting Information). These cracks act as an internal standard for verification of encapsulation, making selection of regions of interest (ROI) straightforward even at relatively low fluences (<1 e^−^/nm^2^). This reduces the requisite pre‐acquisition fluence budget,^[^ ^47^ ^]^ thus allowing more fluence to be used for single frame high resolution imaging or multi‐frame, dynamic time course experiments.

### 2.5 Nutrient Restriction Leads to Enrichment of Different *D.radiodurans* Pleomorphs

As *D.radiourans* demonstrates nutrient‐induced pleomorphisms,^[^ ^30^ ^]^ we grew *D.radiodurans* in nutrient‐restricted conditions (10% v/v LB media) to explore the different ultrastructure of these pleomorphs and to create smaller cell volumes for potential 3D data acquisition using LP‐STEM tomography. It was apparent using light microscopy that nutrient restriction dramatically reduces the number of tetrads and increases the number of monads and diads in solution. This was confirmed in the electron microscope, as most bacteria present were in diad forms. While the diads did appear to have a less dense cytoplasm than tetrads there was little difference in the ultrastructural features, with the primary difference being a more spherical rather than ellipsoidal shape. Interestingly, in some individuals it was clear that the peptidoglycan wall between two individuals was in a ring shape rather than a singular partition suggesting that there may be intercell communication in these diads at this stage of growth (Figure 5B). We also observed diads which do not display this ring‐shaped aperture and instead had a more typical peptidoglycan curtaining partition, these appeared more ellipsoidal, suggesting that there may be a need for this type of aperture at this specific stage of cell growth. A similar decrease in the ellipticity of the cells at specific growth phases has been observed previously using high resolution light microscopy.^[^ ^41^ ^]^

## 3 Conclusion

The LP‐STEM studies presented here demonstrate an effective technique for imaging whole biological specimens and their internal ultrastructure in native aqueous environments, without the need for heavy‐metal staining, nanoparticle tagging or cryogenic/plastic preservation, all of which require specialized equipment and can lead to potential artefacts. These graphene liquid cells, prepared from simple materials, allow for high contrast imaging in LP‐EM analysis. In combination with STEM‐EDX techniques, this method can provide new internal information not available in other native preparation approaches, such as conventional whole‐cell cryo‐transmission electron microscopy/tomography techniques, where samples include layers of vitreous ice.

As the graphene liquid cells are prepared on EM grids, they fit standard EM holders. Unlike commercially available silicon nitride chip‐based liquid cells, the nature of GLCs means each graphene blister is an independent liquid cell, keeping potential radiolysis products from the electron beam localized to a single blister and preventing the build‐up and diffusion of free radicals from causing damage in adjacent cells. The isolated nature of graphene blisters also means that in the event of a failure of a single liquid cell, there is no significant release of liquid inside the microscope column. This contrasts with conventional silicon nitride chip liquid cell constructs where great care is required to ensure the liquid cells remain sealed and failure of the chip may lead to the release of larger volumes of liquid and gases in the column. However, the most significant advance is in imaging quality due to the electron transparency of the graphene and the thinner liquid volume.

There are several variables that we have found to significantly affect sample quality, sensitivity and image SNR. Gold Au‐flat grids produced a tighter encapsulation and more reproducible numbers of blisters, and gold Quantifoil grids gave images with better SNR over larger fields‐of‐view. Buffer composition is also a significant factor; the use of phosphate buffered saline led to substantial amounts of salt precipitates on the grid obscuring the field‐of‐view. We therefore used a compositionally simpler zwitterionic buffer, HEPES, for our experiments. Degassing before encapsulation was also found to be essential to limit the formation of gas bubbles in the buffer under the electron beam.

While *D.radiodurans* is not the only biological specimen imageable using GLCs, our study emphasizes that it has proven to be an extremely valuable test sample for exploring LP‐EM/EDX capabilities. Due primarily to its radiation resistance, elementally heterogeneous nature and round coccoid shape, *D.radiodurans* lends itself readily to study in GLCs. Previously, it was shown that in transcriptionally active *E.coli*, 50% of the population received a lethal fluence (LF_50_) at ≈10 e^−^/nm^2^, in commercially available silicon nitride chips.^[^ ^42^ ^]^ *D.radiodurans* is known to tolerate fluences at least tenfold greater in magnitude than *E.coli*, suggesting an LF_50_ of *D.radiodurans* under the same circumstances of ≈100 e^−^/nm^2^. This fluence limit is further increased by the 2–7x protection factor afforded by graphene encapsulation,^[^ ^48^, ^49^, ^50^ ^]^ leading the way toward low fluence live‐cell imaging in LP‐STEM of other more commonly studied cells.

Our observations of proteins at ≈24 nm and other biomolecules within these bacterial cells suggest that even at cumulative fluences significantly higher than the putative LF_50_ of *D.radiodurans*, it may still be more favorable to monitor protein dynamics and interactions in situ than in purified protein solutions, with beam‐related damage potentially mitigated by the native reactive oxygen defense system of *D.radiodurans*. *D.radiodurans* response to desiccation by creating cracks in their membrane from rapid depressurization in the microscope vacuum, also demonstrates their utility in LPEM analysis, by acting as an internal marker for aqueous EM environments.

We have also demonstrated the use of LP‐STEM‐EDX applications to build an elemental understanding of the bacterial cell at the ultrastructural level. LP‐STEM‐EDX has enabled us to demonstrate that Mn ions localise to polyphosphate granules in situ, without the need for denaturants and fixatives. We have also mapped significant concentrations of sulfur residing in the periplasm, suggesting a potential mechanism for oxidative homeostasis in *D.radiodurans*. This periplasmic sulfur may help manage redox homeostasis within *D.radiodurans*, by being released into the cytoplasm on irradiation to help inhibit oxidative damage induced by free radicals and protect biomolecules critical to cellular survival. These LP‐STEM‐EDX studies demonstrate the potential to use both native elemental distributions and non‐native elemental probes to localize biomolecules and structures of interest within a cell.

In conclusion, this study serves as a proof of principle demonstrating how LP‐STEM‐EDX enables studies of large‐scale cellular events, such as cell division, under relatively low fluence conditions (<100 e^−^/nm^2^) and can also resolve even fine ultrastructural details, such as proteins albeit at a higher fluence. The techniques described herein illustrate the potential of LP‐EM to explore fundamental biological questions in situ. Electron fluence, sample thickness and the fundamental characteristics of graphene, such as its grain size, may however limit its broader application to significantly larger cells and tissues. Ultimately, LP‐EM techniques combined with emerging low‐fluence imaging applications such as ptychography^[^ ^51^, ^52^ ^]^ and compressive sensing^[^ ^53^ ^]^ mean that imaging cellular dynamics in vivo at sub‐nanometer resolutions in an electron microscope may soon be achievable in many cellular systems.

## 4 Experimental Section

### Growth of D. radiodurans

Five ml of Lysogeny Broth (LB) [1% w/v Glucose] was inoculated with *D. radiodurans* (Strain R_1_; DSMZ 20 539) frozen culture and incubated at 30 °C for 24 h with shaking. 500 µl of the resulting culture was added to 5 ml of fresh LB [1% w/v Glucose] and after this reached early exponential phase (OD_600_: ≈0.3) was further incubated and then collected at different time points: 15 min (early exponential phase, A600 nm = 0.35), 2 h (mid‐exponential phase, A600 nm = 0.65) and 20 h (stationary phase, A600 nm = 1.65). For the Mn accumulation experiments, early exponential phase bacteria were incubated with sterile‐filtered MnCl_2_ [0.1 or 1 mм] for 4 h, then spun down and encapsulated. A control growth with water was performed and followed simultaneously. For the restrictive media experiment, 500 µl of stationary phase bacteria were added to 5 mL of fresh 10% LB media in ddH_2_O [1% w/v Glucose] and incubated overnight. 2 ml of the resulting samples were then spun down at either 500 g for the LB preparations and 2000 g for the restrictive preparations for 5 min, resuspended in 1 ml of nitrogen degassed 4‐(2‐hydroxyethyl)−1‐piperazineethanesulfonic acid (HEPES) buffer [25 mм] and encapsulated in graphene for LP‐STEM/EDX imaging.

### Brightfield Light Microscopy Imaging of DAPI‐Stained D.radiodurans

Cells were stained with either 4′,6‐diamidino‐2‐phenylindole (DAPI) [36 µм] or 3,3′‐[(3,3′‐dimethylbiphenyl‐4,4′‐diyl)didiazene‐2,1‐diyl]bis(5‐amino‐4‐hydroxynaphthalene‐2,7‐disulfonic acid) (Trypan Blue) [0.2% w/v]. Imaging was carried out on an upright brightfield light microscope, Eclipse Ni (Nikon, USA) using 4x, 10x, and 40x air immersion objectives. Brightfield images were acquired using a 100 W Halogen Lamp with a 100 ms exposure time. LED color channels of wavelength 385 nm (Power: 0.15), 475 nm (Power: 0.65) and 550 nm (Power: 0.35) were used in conjunction with a dichroic mirror EX 377/50 (DM409, Nikon, USA), EX 466/40 (DM495, Nikon, USA) and EX 562/40 (DM 593, Nikon, USA), respectively. Images were collected on a DS‐Fi3 Monochromatic Microscope Camera using NIS‐Elements BR software (Nikon, USA) with a 1s exposure time.

### Graphene Liquid Cell Encapsulation

The hydrated graphene enclosed environments were obtained by first creating a graphene TEM grid. This was performed by transferring graphene (Grolltex, USA) to a TEM grid using cellulose acetate butyrate^[^ ^54^ ^]^ followed by a dry‐cleaning procedure^[^ ^55^ ^]^ at 310 °C overnight. This forms the base of the graphene liquid cell. The top graphene layer for encapsulation was fabricated using a polymer‐free transfer method,^[^ ^56^ ^]^ which provides a flat top surface to the hydrated environment which then adapts to the morphology of the target. The main steps of this process are described in more detail in refs. [26, 57] which were used to prepare the base TEM grid and the creation of the encapsulated environments respectively.^[^ ^58^ ^]^ The commercial based TEM grids used throughout the different experiments to create the hydrated encapsulated environments were Au‐Flat (2/2) or (0.6/1), 300 Mesh, 45 nm thick from Protochips and Au Quantifoil R1.2/1.3 300 mesh from Quantifoil.

### Liquid Phase STEM Imaging

STEM imaging was performed on a 300 kV ARM300 CF 2 (JEOL, Japan) with a spherical aberration corrector, with an illumination convergence semi‐angle (α) of 15.95 mrad and conventional annular dark‐field (ADF) detector [inner collection semi‐angle (β^in^: 37.1 mrad)/outer collection semi‐angle (β^out^: 83 mrad) and bright‐field (BF) detectors (β_BF_: 20.8 mrad). The optics were aligned using a standard gold waffle thin film. Images of aqueous bacteria were typically recorded at 20 kX, 100 kX, and 200 kX magnifications (pixel size: 5, 1 and 0.5 nm respectively), 2048 × 2048 frame size and 20 µs dwell time.

### Liquid Phase EDX Imaging

Energy‐dispersive X‐ray spectroscopy was performed in STEM mode on the instrument above (JEOL, Japan) on aqueous bacterial samples at 20kX, 100 kX and 200 kX magnifications (pixel size: 20, 4, and 2 nm) respectively), 512 × 512 frame size and 20 µs dwell times, using dual liquid N_2_ cooled Si (Li) JEOL detectors with a combined solid angle of 1.41 sr.

### STEM/EDX Image Post‐Processing

To enhance high‐resolution details and to remove background noise, a Gaussian bandpass filter was applied to each micrograph (High Pass:300 px/Low Pass:2 px), using the ImageJ Bandpass filter plugin. Horizontal STEM scanning artefacts were smoothed using the “Suppress Stripes” function at 5% in the ImageJ Bandpass filter plugin. All images were then normalized to 0.35% pixel saturation, using the “Contrast Enhance” function in ImageJ.

EDX maps were produced using the JEOL Analysis Station Software (JEOL, Japan) and processed in Hyperspy.^[^ ^59^ ^]^ Maps were binned by 2 in x‐y and Gaussian filtered (σ = 2 px) to reduce background, using the “Gaussian Blur” function in ImageJ. All maps were then contrast normalized and equalised to 0.35% pixel saturation, using the “Contrast Enhance” function in ImageJ. All maps were generated from the respective elemental Kα lines.

### Fluence History Tracking with Axon Studio

The Axon Studio software from Protochips Inc., was used in conjunction with Axon Dose to accurately track the fluence history of each imaged area, providing an accurate readout of cumulative specimen fluence.

## Conflict of Interest

The authors declare no conflict of interest.

## Supporting information

Supporting Information

## Data Availability

The data that support the findings of this study are openly available in Zenodo at 10.5281/zenodo.10625794 and 10.5281/zenodo.11527417.
