# Supplementary material for: Liquid Phase Electron Microscopy of Bacterial Ultrastructure
Source: Small. 2024 Sep 6;20(50):2402871. doi: 10.1002/smll.202402871 (PMC11636060; doi:10.1002/smll.202402871)
Supplement: Supplementary file 1 — Supporting Information [file SMLL-20-2402871-s001.docx]

Supporting Information

**Liquid Phase Electron Microscopy of Bacterial Ultrastructure**

*Brian J. Caffrey^+*^, Adrián Pedrazo-Tardajos^+^, Emanuela Liberti, Benjamin Gaunt, Judy S. Kim^*^ and Angus I. Kirkland.*

^+^ Equal contribution

^*^ Corresponding authors: brian.caffrey@rfi.ac.uk

judy.kim@materials.ox.ac.uk

**Analysis of sustained pressures inside graphene encapsulated** *D.radiodurans*

We have measured the average size of the *D.radiodurans* tetrads*.* Based on the measurements from 13 bacteria from ADF-STEM images, *D.radiodurans* has an ellipsoidal geometry. The average lengths of the long axis **(A)** and short axis **(B)** are 2.91 ± 0.23 µm and 2.09 ± 0.13 µm, respectively and median of 2.80 μm and 2.08 μm. These values include both random and systematic errors. The standard deviations of the lengths of the long axis and short axis are 0.57 µm and 0.32 µm, respectively (Fig.S4).

The pressure inside a graphene liquid cell can be estimated by using the pressure of Laplace^1^:

$$\Delta P=\frac{\gamma}{2R}$$

Where *ΔP* is the Laplace pressure gradient, γ the interfacial energy of graphene−water and *R* is the radius of the encapsulated *D.radiodurans*. The error associated with the calculated value is:

$$\left( E \right)^{2}=\left( \frac{\partial f(X_{1},X_{2},\ldots)}{\partial X_{1}}\Delta X_{1} \right)^{2}+\left( \frac{\partial f(X_{1},X_{2},\ldots)}{\partial X_{2}}\Delta X_{2} \right)^{2}+\ldots.,$$

Thus:

$$E_{\Delta P}=\frac{\gamma\cdot E_{R}}{2R^{2}}$$

$$\Delta P_{1}=\frac{\gamma_{Graphene-Water}}{2R_{1}}=\frac{0.09\frac{J}{m^{2}}}{2\cdot1.05\cdot{10}^{-6} m}=\left( 4.3\pm0.5 \right){\cdot10}^{+4}\frac{J}{m^{3}}=0.42 \pm0.05 atm$$

$$\Delta P_{2}=\frac{\gamma_{Graphene-Water}}{2R_{2}}=\frac{0.09\frac{J}{m^{2}}}{2\cdot1.46\cdot{10}^{-6} m}=\left( 3.1\pm0.5 \right){\cdot10}^{+4}\frac{J}{m^{3}}=0.31\pm0.05 atm$$

To calculate the pressure exerted by graphene directly, without intermediate water, on a hydrated *D.radiodurans*, some approximations can be applied. The first is that the membrane of a *D.radiodurans* is chemically similar to that of *E.coli*, and hence the surface tension can be estimated. Therefore, we can use the measured interaction force between a single flake of pristine graphene and the cell wall of a living *E.coli*: 38.2 ± 16.4 pN.^2^ Then, using the following equation the interfacial energy can be approximated:

$$\gamma=\frac{F}{L}$$

Where *F* is the adhesive force and *L* is length over which that force is distributed. In the case of *D.radiodurans* we can simplify its geometry to a circumference, where *d* is the diameter of the *D.radiodurans* which value is in the range of length of the long and short axis previously measured (2.1-2.9 μm):

$$\gamma_{1}=\frac{F}{\pi\cdot d}=\frac{38.2\cdot{10}^{-12}N}{\pi\cdot2.1 \cdot{10}^{-6} m}=\frac{38.2\cdot{10}^{-6}N}{\pi\cdot2.1 m}=5.8\cdot{10}^{-6}\frac{J}{m^{2}}$$

$$\gamma_{2}=\frac{F}{\pi\cdot d}=\frac{38.2\cdot{10}^{-12}N}{\pi\cdot2.9\cdot{10}^{-6} m}=\frac{38.2\cdot{10}^{-6}N}{\pi\cdot2.9 m}=4.2\cdot{10}^{-6}\frac{J}{m^{2}}$$

The error associated can be calculated using the same equation for the error used in the pressure calculations, hence:

$$E_{\gamma}=\sqrt{\left( \frac{E_{F}}{L} \right)^{2}+\left( \frac{F\cdot E_{L}}{L^{2}} \right)^{2}}$$

$$E_{\gamma_{1}}=\sqrt{\left( \frac{16.4\cdot{10}^{-6}}{\pi\cdot2.1} \right)^{2}+\left( \frac{16.4\cdot{10}^{-12}\cdot0.1\cdot{10}^{-6}}{\left( 2.1\cdot{10}^{-6} \right)^{2}} \right)^{2}}=2.5\cdot{10}^{-6}\frac{J}{m^{2}}$$

$$E_{\gamma_{2}}=\sqrt{\left( \frac{16.4\cdot{10}^{-6}}{\pi\cdot2.9} \right)^{2}+\left( \frac{16.4\cdot{10}^{-12}\cdot0.2}{{2.9}^{2}} \right)^{2}}=1.8\cdot{10}^{-6}\frac{J}{m^{2}}$$

$$\gamma_{1}=(5.8\pm2.5)\cdot{10}^{-6}\frac{J}{m^{2}}$$

$$\gamma_{2}=(4.2\pm1.8)\cdot{10}^{-6}\frac{J}{m^{2}}$$

We can estimate the pressure within the encapsulated region from the surface tension exerted by pristine graphene on the membrane surface. This estimation is based on the Laplace pressure equation. The error in this estimation can be calculated:

$$E_{P}=\sqrt{\left( \frac{\Delta\gamma}{2R} \right)^{2}+\left( \frac{\gamma\cdot\Delta R}{2R^{2}} \right)^{2}}$$

$$\Delta P_{1}=\frac{\gamma_{1}}{2R_{1}}=\frac{5.8\cdot{10}^{-6}\frac{J}{m^{2}}}{2\cdot1.05\cdot{10}^{-6} m}=2.76 \pm1.23\frac{J}{m^{3}}=(2.7\pm1.2)\cdot{10}^{-5}atm$$

$$\Delta P_{2}=\frac{\gamma_{2}}{2R_{2}}=\frac{4.2\cdot{10}^{-6}\frac{J}{m^{2}}}{2\cdot1.45\cdot{10}^{-6} m}=1.45\pm0.66\frac{J}{m^{3}}=(1.4\pm0.7)\cdot{10}^{-5} atm$$

This pressure can be compared with the pressure calculated using a more detailed analysis which includes other pressure components such as the Van der Waals and elastic, from Khestanova *et al*^3^ who derived the following relationship:

$$P=\frac{4\pi\gamma}{5\cdot1.7}\left( \frac{5c_{1}Y}{\pi\gamma} \right)^{\frac{1}{6}}\left( \frac{1}{t\cdot l^{2}} \right)^{\frac{1}{3}}$$

Where $\gamma$is the total adhesion energy ($\gamma=\gamma_{Graphene-Graphene}-\gamma_{Graphene-Water}$, where $\gamma_{Graphene-Graphene}$ and $\gamma_{Graphene-Water}$are the adhesion energy between two graphene layers and graphene and water respectively), $Y$ is the Young’s modulus and $t$ and $l$ are the height and the radius of the encapsulated area, respectively. Substituting the variables with our values we reach almost twice atmospheric pressure:

$$P=161389.4 Pa=1.59 atm$$

This equation can also be used to estimate the total pressure of other encapsulated biological structures, such as viruses and proteins in future experiments (Fig. S5).


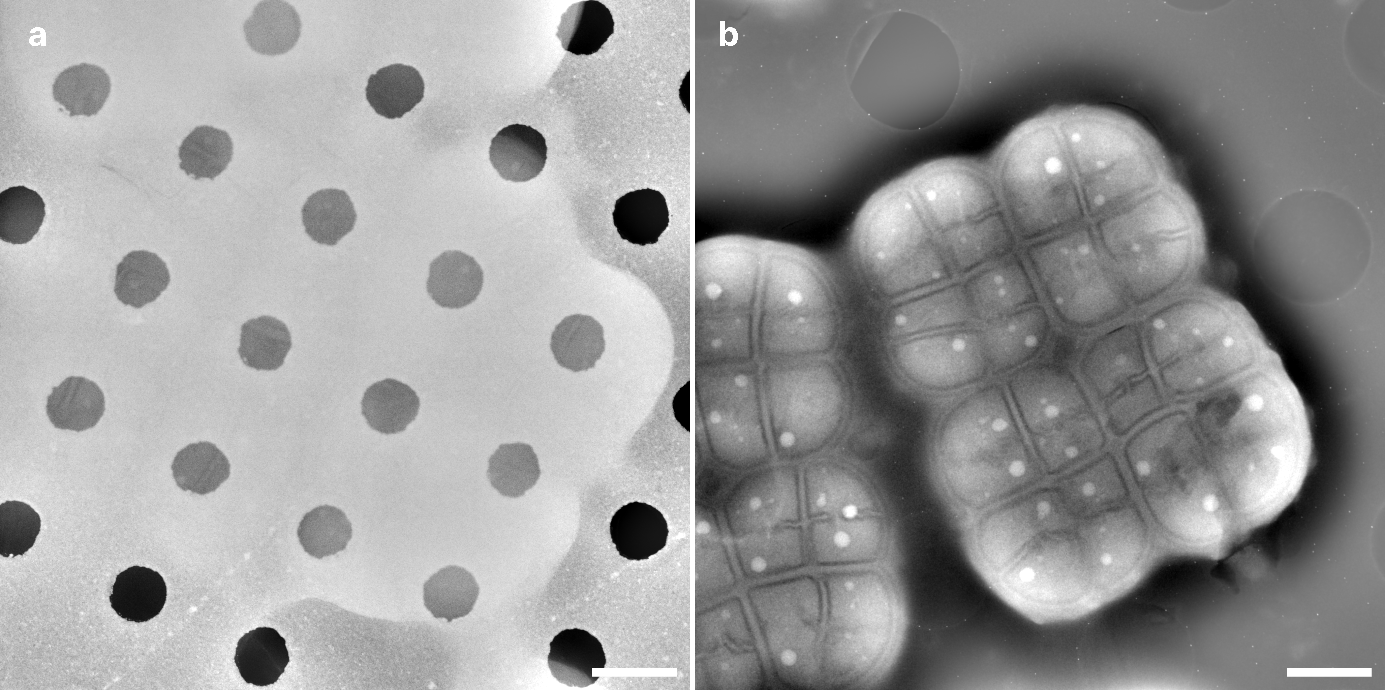


**Figure S1.** Comparison between different grid substrates.

a) LP-ADF-STEM images of late stationary stage (OD_600_: 1.65) *D.radiodurans* encapsulated on 0.6 µm hole Au-Flat and b) the same culture encapsulated on 1.2 µm hole Au Quantifoil grids, which have a holey carbon film.


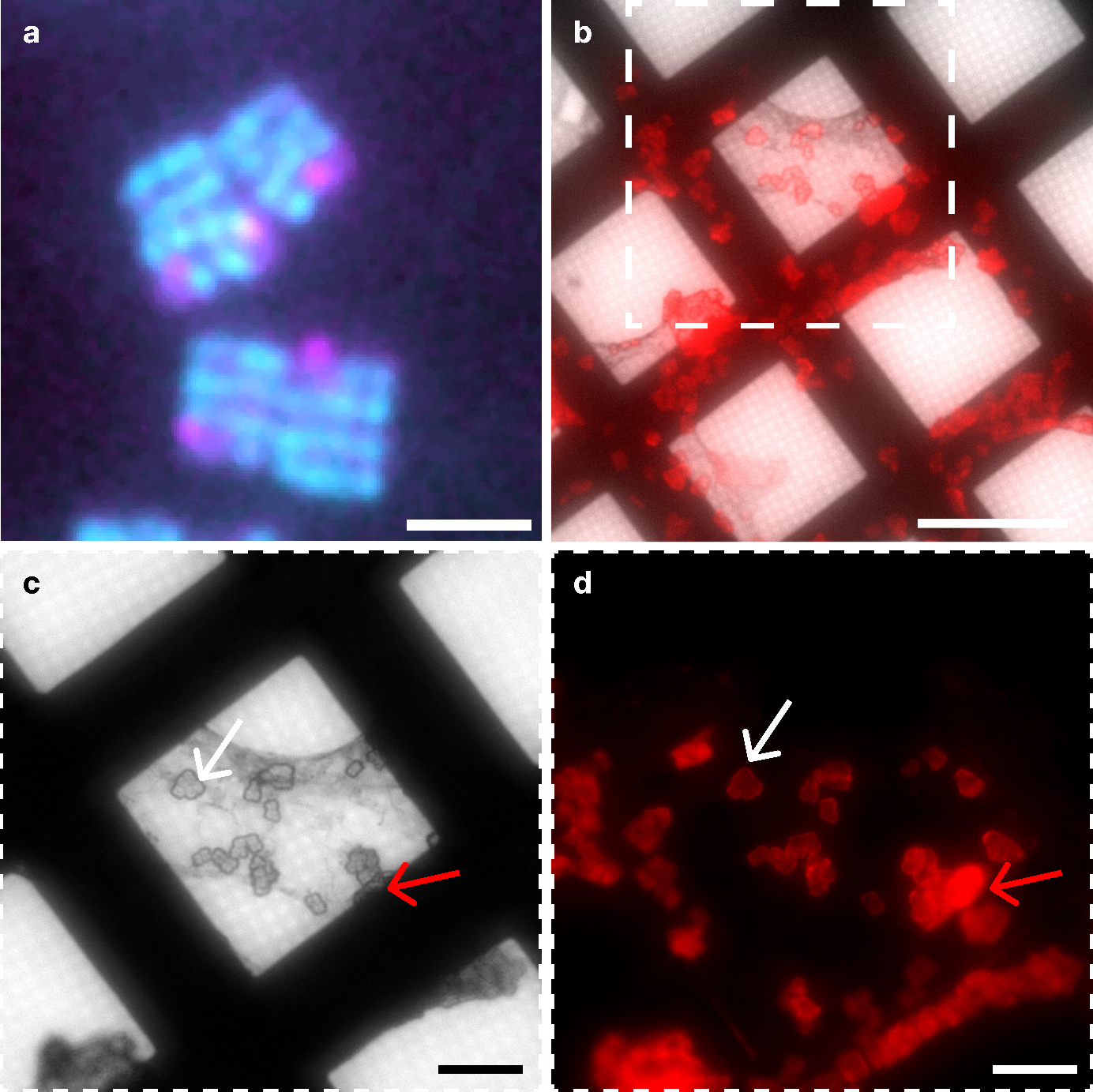


**Figure S2.** LM analysis of *D.radiodurans*.

a) Fluorescence Light Microscopy image of DAPI-stained *D.radiodurans* grown in LB broth, showing nucleoid (cyan) and phosphate granules (magenta). b) Combined brightfield and fluorescence image of *D.radiodurans* encapsulated with Trypan Blue (TB). c) Zoom into ROI indicated in (b) and d) corresponding TB fluorescence stain. White Arrow: TB excluded/Membrane intact. Red arrow: TB stained/Membrane ruptured; Scale bar: a: 5 µm; b: 50 µm; c,d: 20 µm.


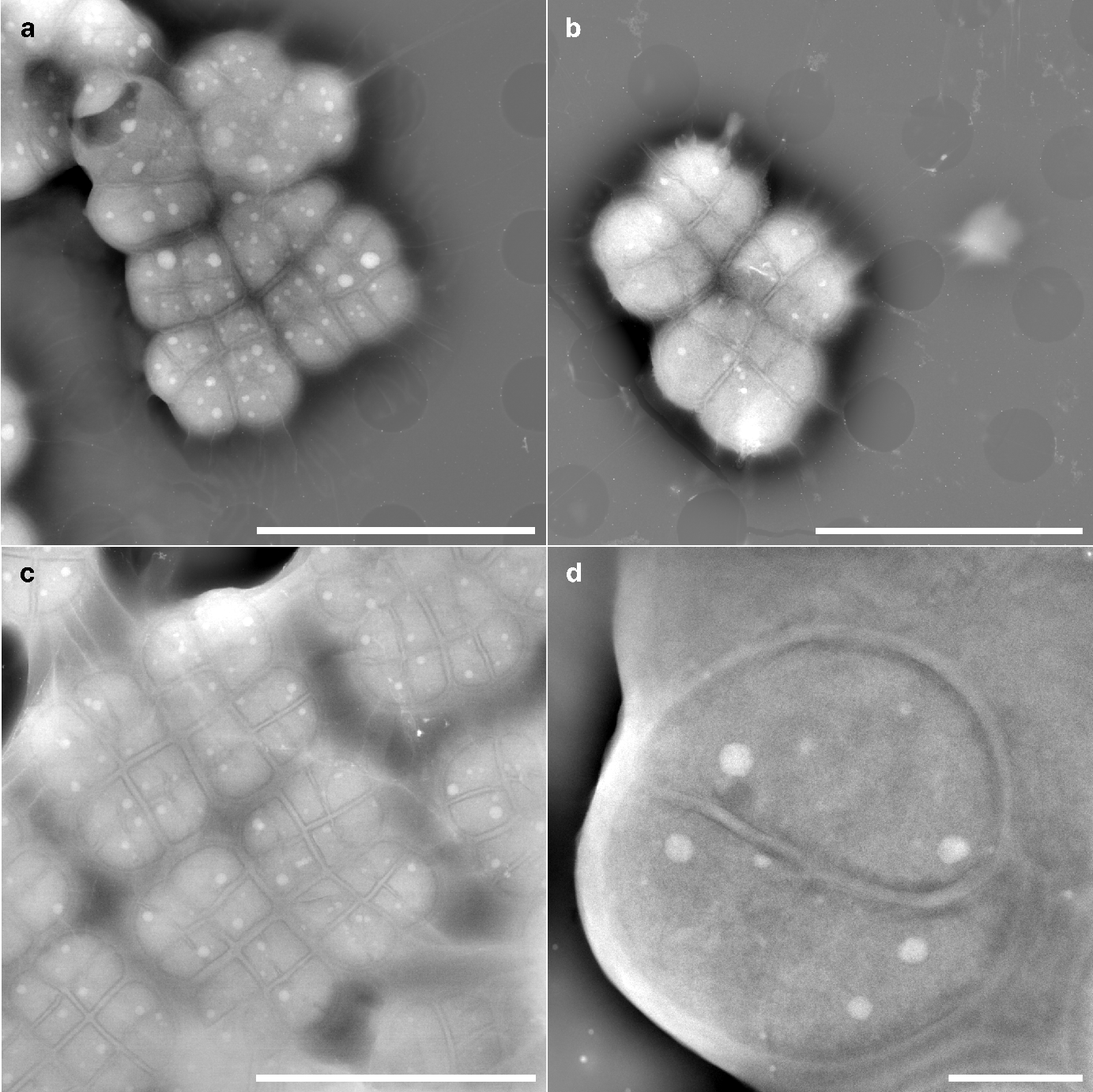


**Figure S3** LP-ADF-STEM image of encapsulated *D.radiodurans* growth phases.

a) Early exponential phase (OD_600_: 0.35), b) mid-exponential phase (OD_600_: 0.65) and c) late stationary phase (OD_600_:1.65). d) LP-ADF-STEM image of encapsulated *D.radiodurans* diad from the late stationary phase sample. (TCF: 93 e^-^/nm^2^) Scale bar: a-c: 5 µm. d: 500 nm.


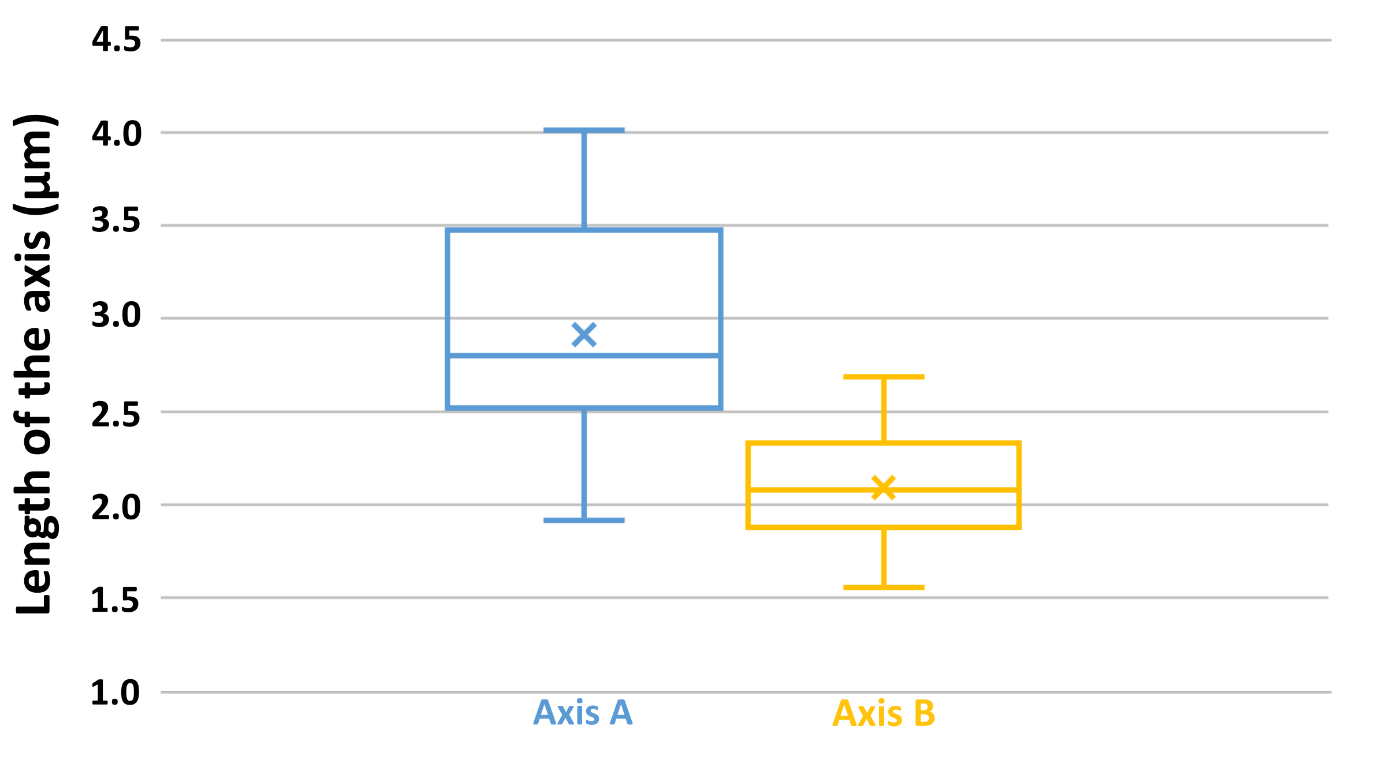


**Figure S4.** *D.radiodurans* size distribution.

The center line indicates the median values (Median of 2.80 μm and 2.08 μm); × indicates the mean (2.91 ± 0.57 μm and 2.09 ± 0.32 μm).


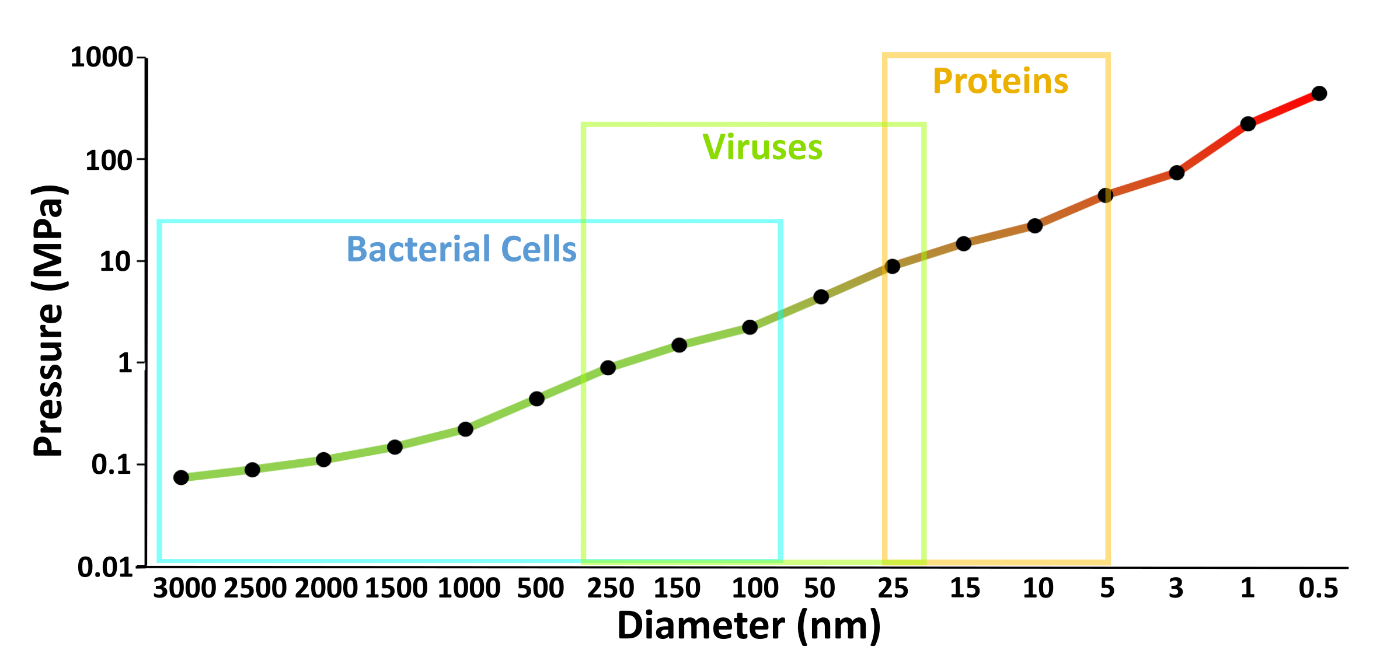


**Figure S5.** Pressure in different volumes of spherical, graphene-encapsulated structures and its correlation with the size of relevant biological structures.

**
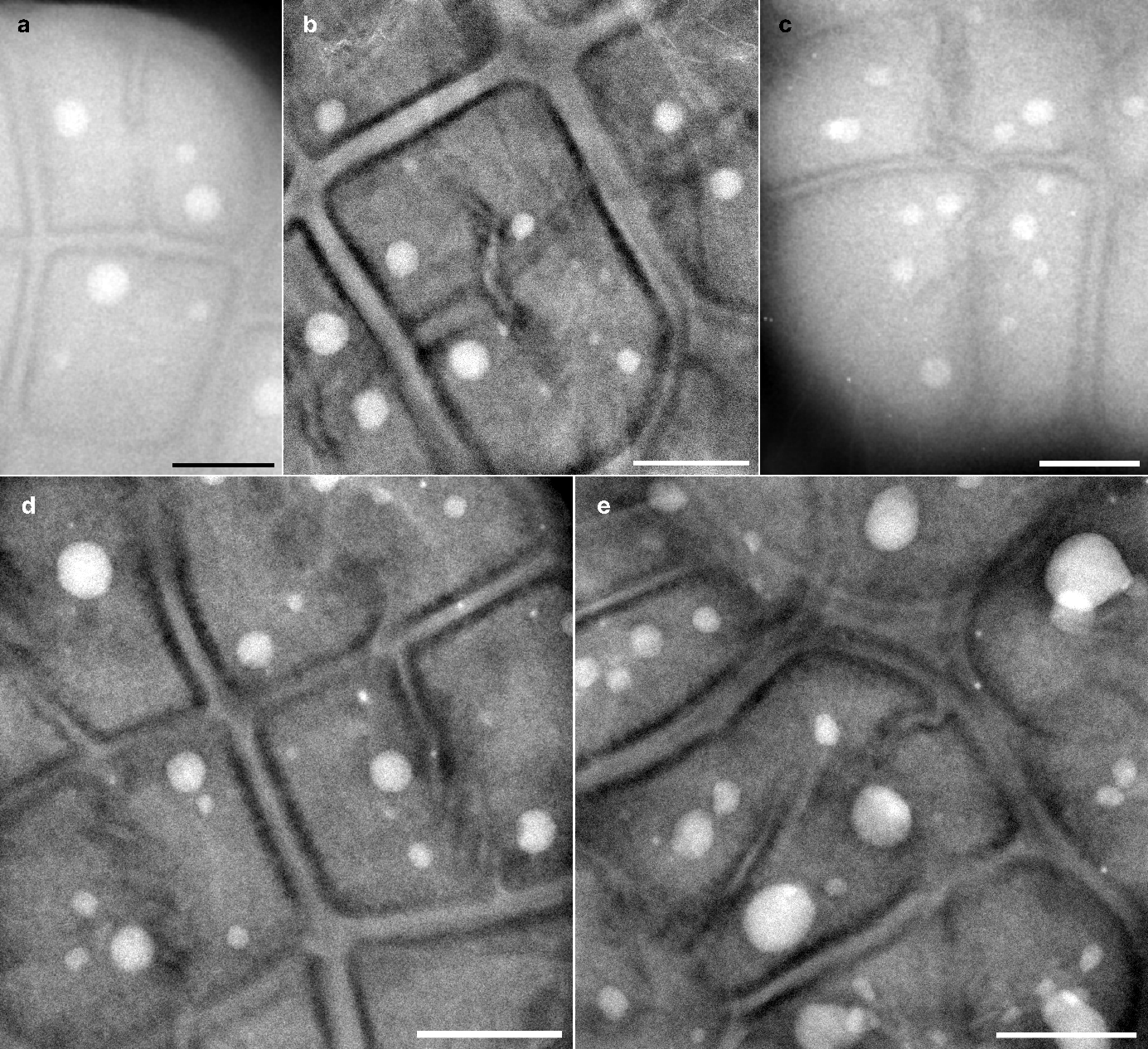
**

**Figure S6.** Asymmetric division and other irregularities in septal development in *D.radiodurans*.

a) Individual cocci within a tetrad at different stages of cell division (Late stationary phase). b) S-shaped septum without complete perpendicular septum (Late stationary phase). c) S-shaped septum with complete perpendicular septum (Early exponential phase**).** d) Diagonal septa within individual cocci in late stationary phase and (e) Early exponential phase. Scale bar: 500 nm.


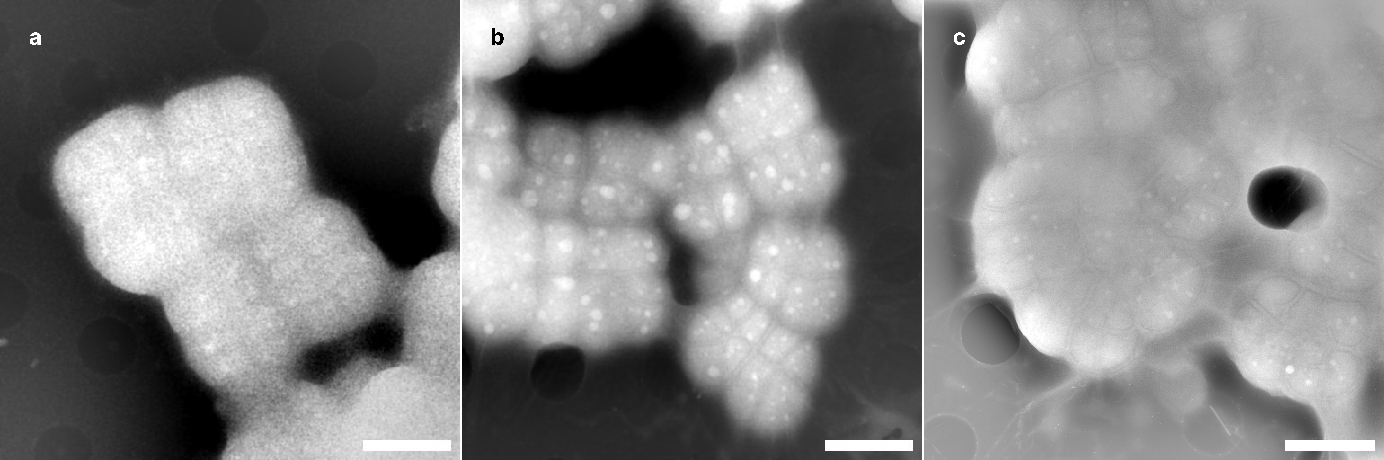


**Figure S7.** *D.radiodurans* imaged at different fluence.

BP-filtered LP-ADF-STEM images of *D.radiodurans* at fluences of a) 0.5, b) 5.8 and c) 65 e/nm^2^. Individual cocci and endogenous polyphosphates are clearly visible at fluences as low as 5.8 e^-^/nm^2^. Scale bar: 2 µm.

**
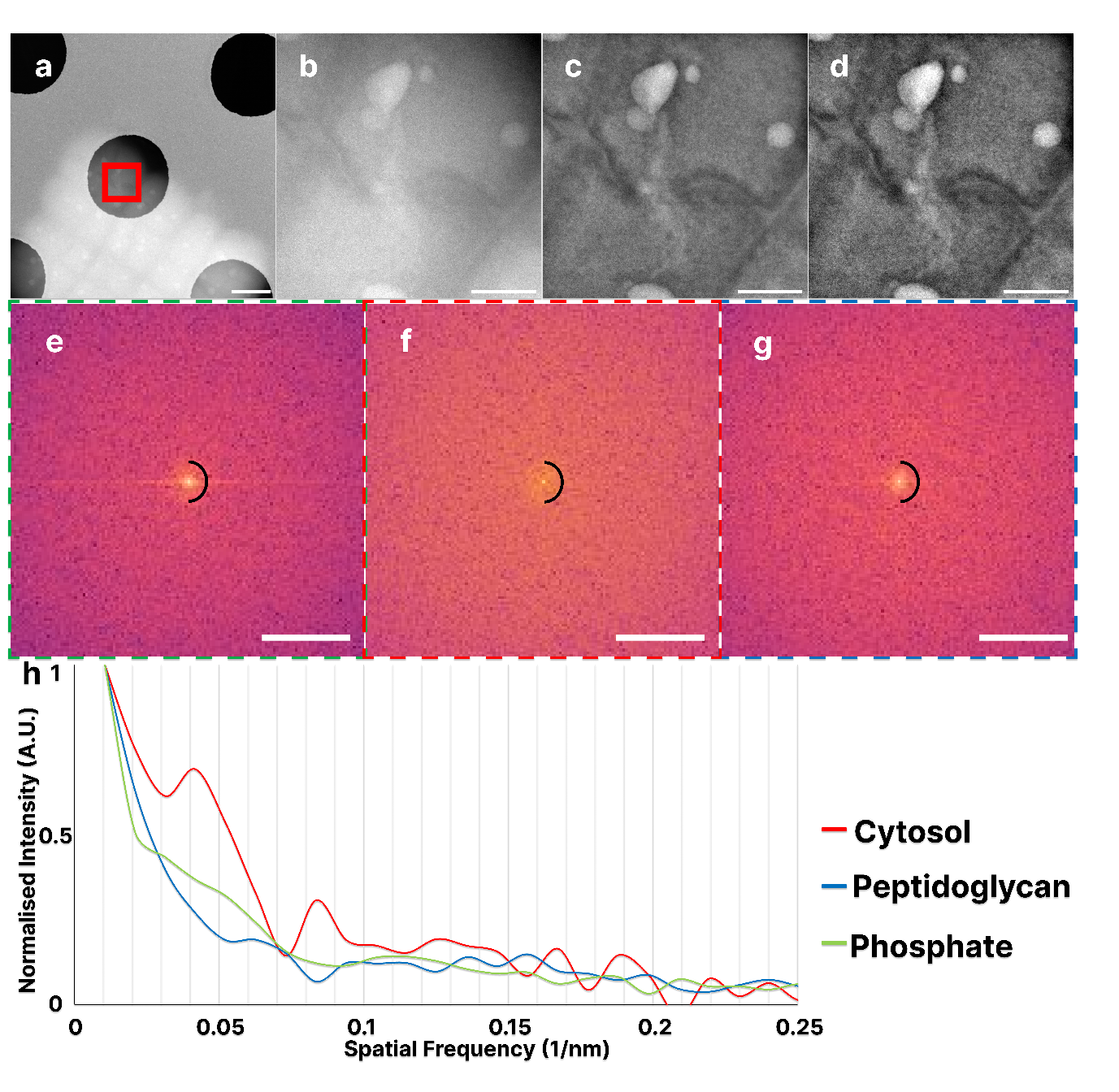
**

**Figure S8.** *D.radiodurans* ultrastructure in graphene encapsulated environments.

a) Low magnification ADF-STEM image of an encapsulated group of *D.radiodurans* tetrad, Fig.3A ROI marked in red. b) Raw ADF-STEM image of *D.radiodurans*, c) After bandpass filter applied to (b), d) after contrast enhancement to (c). Corresponding pseudo-colored expanded power spectra for ROIs in Figure 3b of storage granules (e), cytosol (f; Figure 3b inset) and peptidoglycan (g), (18 nm)^-1^ boundary marked by semicircles. h) Radial profile plot of the power spectra (e-g), demonstrating a peak frequency ~0.0417 nm^-1^ in the cytosol not present in the peptidoglycan. Scale bar: a: 1 µm; b-d: 250 nm; PS: (4 nm)^-1^.

**
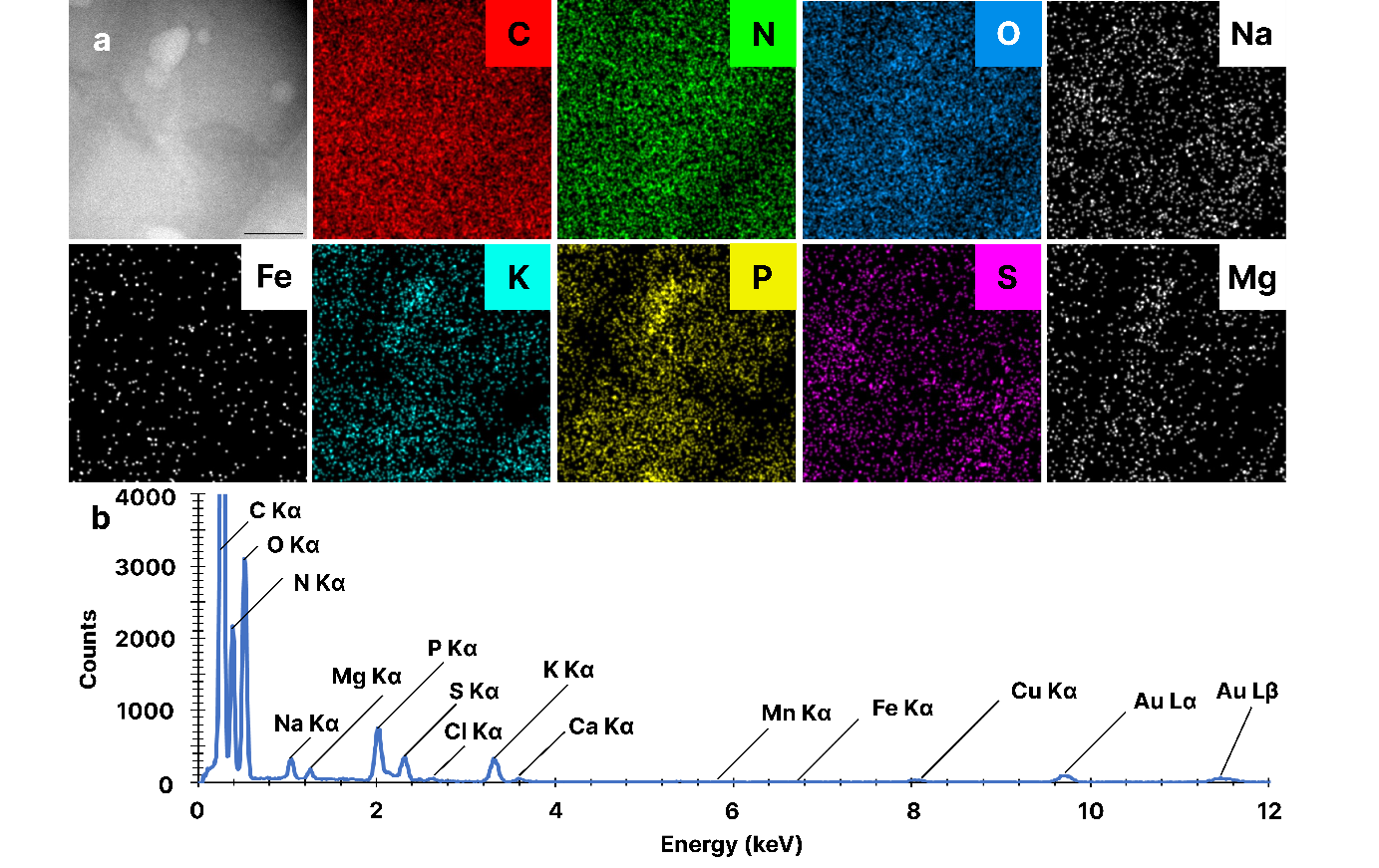
**
**Figure S9.** *D.radiodurans* elemental distribution in graphene encapsulated environments.

a) Raw ADF-STEM image taken from Figure 3a and individual element maps. b) EDX spectrum of (a) with no signal evident for Fe Kα lines. Scale bar: a: 250 nm.


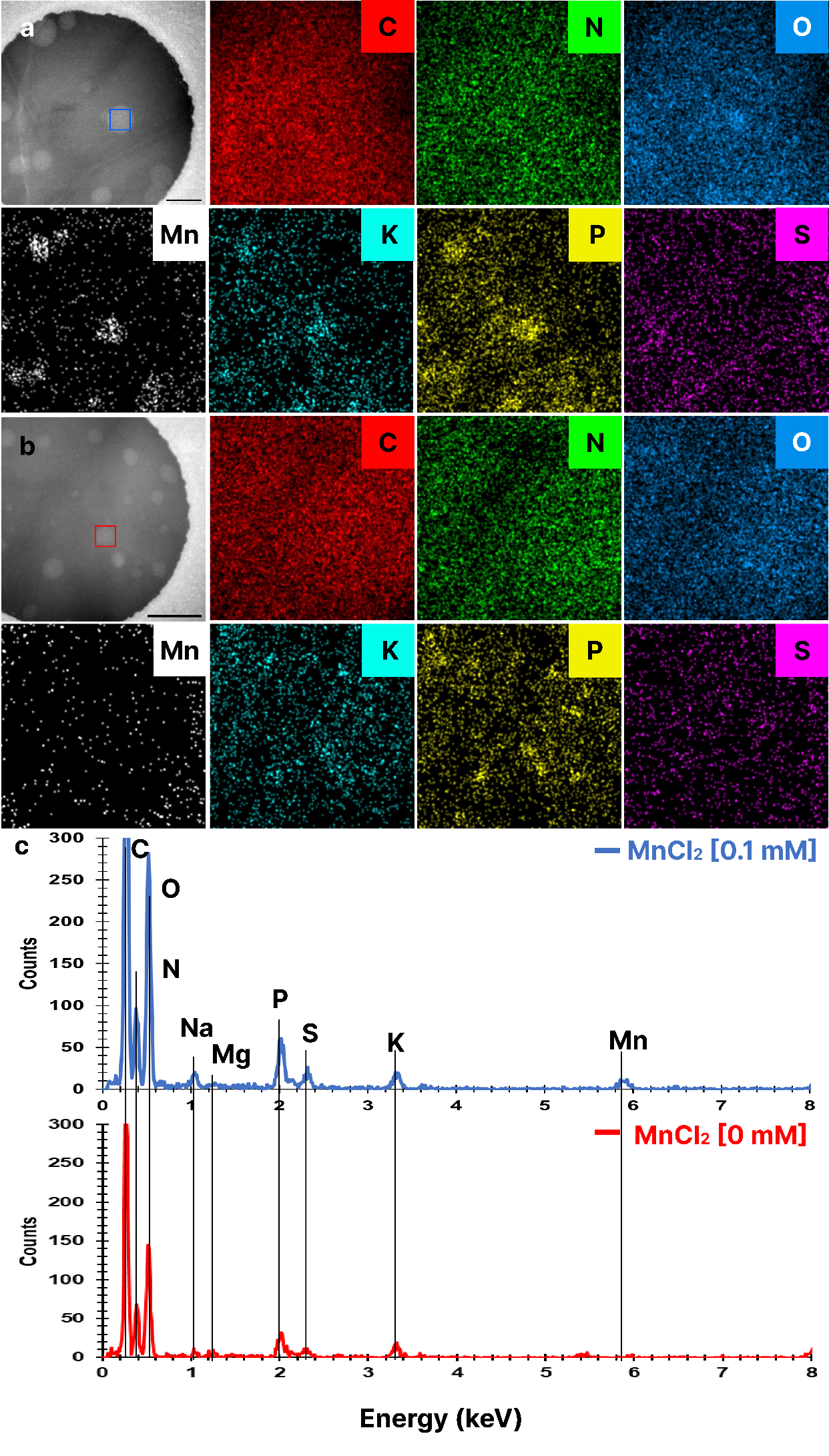


**Figure S10.** Manganese accumulation in bacterial phosphate granules.

Individual element maps of *D.radiodurans* treated with (a) and without (b) MnCl_2_. c) EDX spectra of individual phosphate granules, selected area (200 x 200 nm^2^) marked in (a) and (b). Scale bar: a,b: 500 nm.


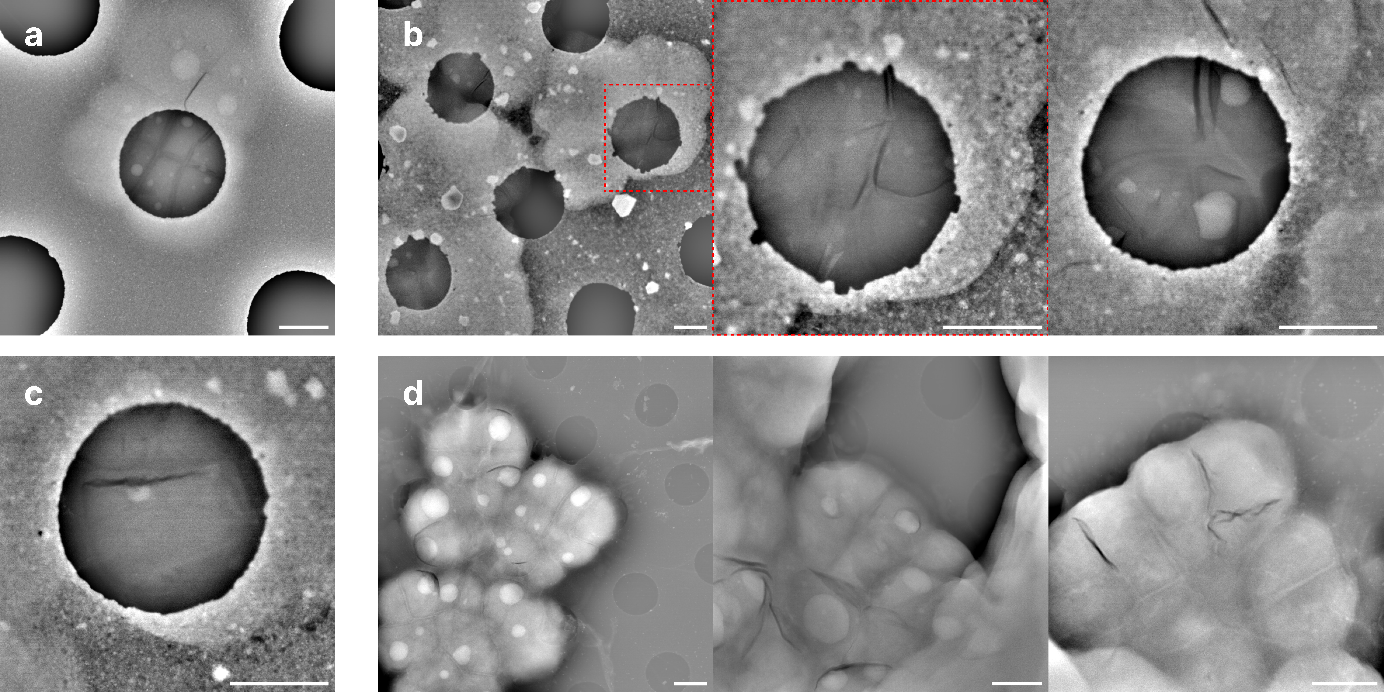


**Figure S11.** Further examples of vacuum desiccation in torn graphene liquid cells.

a-d) LP-ADF-STEM images of vacuum desiccated bacteria, showing the membrane cracks, from four independent experiments. Scale bar: 1 µm

**References:**

1. Ghodsi, S.M., Sharifi-Asl, S., Rehak, P., Král, P., Megaridis, C.M., Shahbazian-Yassar, R., and Shokuhfar, T. (2020). Assessment of Pressure and Density of Confined Water in Graphene Liquid Cells. Adv. Mater. Interfaces. https://doi.org/10.1002/admi.201901727.

2. Al Faouri, R., Henry, R., Biris, A.S., Sleezer, R., and Salamo, G.J. (2017). Adhesive force between graphene nanoscale flakes and living biological cells. J. Appl. Toxicol. https://doi.org/10.1002/jat.3478.

3. Khestanova, E., Guinea, F., Fumagalli, L., Geim, A.K., and Grigorieva, I. V. (2016). Universal shape and pressure inside bubbles appearing in van der Waals heterostructures. Nat. Commun. https://doi.org/10.1038/ncomms12587.
